# Using the Biocheck.UGent™ scoring tool in Irish farrow-to-finish pig farms: assessing biosecurity and its relation to productive performance

**DOI:** 10.1186/s40813-018-0113-6

**Published:** 2019-03-01

**Authors:** Maria Rodrigues da Costa, Josep Gasa, Julia Adriana Calderón Díaz, Merel Postma, Jeroen Dewulf, Gerard McCutcheon, Edgar Garcia Manzanilla

**Affiliations:** 1 Pig Development Department, Teagasc - Animal & Grassland Research and Innovation Centre, Moorepark, Fermoy, Co. Cork, Ireland; 2 Departament de Ciència Animal i dels Aliments, Facultat de Veterinaria, Universitat Autònoma de Barcelona, Bellaterra, 08193 Barcelona, Spain; 3 Department of Reproduction, Obstetrics and Herd Health, Veterinary Epidemiology Unit, Faculty of Veterinary Medicine, Ghent University, Salisburylaan 133, 9820 Merelbeke, Belgium; 4 Pig Development Department, Teagasc Oak Park, Co. Carlow, Ireland; 5 School of Veterinary Medicine, University College Dublin, Belfield, Dublin 4, Ireland

**Keywords:** Biosecurity, Efficiency, Ireland, Productive performance, Swine production

## Abstract

**Background:**

Biosecurity is one of the main factors affecting disease occurrence and antimicrobial use, and it is associated with performance in pig production. However, the importance of specific measures could vary depending on the (national) context. The aim of this study was to describe the biosecurity status in a cohort of Irish pig farms, to investigate which of those biosecurity aspects are more relevant by using the Biocheck.UGent™ scoring system, and to study the impact of such aspects on farm performance.

**Results:**

External biosecurity score was high compared to most countries due to the characteristics of the Irish pig sector (i.e. purchasing only semen and breeding gilts on farm). The internal biosecurity score was lower and had greater variability among farms than other EU countries. Using multivariable linear regression, the biosecurity practices explained 8, 23, and 16% of variability in piglet mortality, finisher mortality, and average daily gain, respectively. Three clusters of farms were defined based on their biosecurity scores (0 to 100) using principal components and hierarchical clustering analysis. Scores for clusters 1, 2 and 3 were (mean ± SD) 38 ± 7.6, 61 ± 7.0 and 66 ± 9.8 for internal and 73 ± 5.1, 74 ± 5.3 and 86 ± 4.5 for external biosecurity. Cluster 3 had lower piglet mortality (*P =* 0.022) and higher average daily gain (*P =* 0.037) when compared to cluster 2.

**Conclusions:**

Irish farms follow European tendencies with internal biosecurity posing as the biggest liability. Our results suggest that practices related to the environment and region, feed, water and equipment supply, and the management of the different stages, need to be addressed in lower performing farms to improve productive performance. Further studies on the economic impact of these biosecurity practices including complementary data on herd health, gilt rearing, piglet management, vaccination and feeding strategies are needed.

## Background

Pig production is the third biggest agricultural activity in Ireland [1], with a self-sufficiency of 219% [2]. In 2016, there were approximately 150,000 breeding sows, producing an estimate of 4,000,000 pigs per year [2]. The Irish pig sector has identified animal health and management as key inputs for productivity, and highlighted that the biggest challenges in the Irish setting are the control of endemic diseases, the prevention of introduction of exotic diseases, and the reduction of the use of antimicrobials, among others [1]. In the last few years, biosecurity practices have been widely discussed. On-farm biosecurity protects farms from disease by preventing pathogenic agents to enter (external biosecurity) or spreading once inside the farm (internal biosecurity, which can also overlap with management). Good biosecurity practices were related to improved performance, better financial return for farmers [3, 4], and to a low use of antimicrobials [5, 6]. Moreover, few studies provide quantitative data effectively linking production performance to biosecurity [5, 7, 8]. The Biocheck.UGent™ scoring system developed by Gent University [9] assesses biosecurity using a risk assessment approach and it has been successfully applied in several EU countries [3, 10, 11]. Postma et al. [3] showed that biosecurity has moderate correlations to production performance in four European countries (Belgium, France, Germany, Sweden) and concluded that biosecurity practices vary with the country. This indicates that characterization and contextualization of the pig sector in each national setting are important to understand the connection between biosecurity and performance [11]. For research purposes, this contextualization is difficult when the methods used across countries are not the same. However, record keeping and benchmarking within and between countries are necessary tools for an efficient and competitive sector. In this study, we aimed to describe biosecurity status in Irish pig farms, to investigate which biosecurity aspects were more relevant by using the Biocheck.UGent™ scoring system, and to study the impact of such aspects on farm performance.

## Methods

Fifty-eight Irish pig farms were scored between February and May 2016 using the biosecurity scoring system Biocheck.UGent™. Performance data for 2016 for these farms were retrieved from Teagasc e-Profit Monitor (ePM) – a national herd monitoring system – and the effect of the biosecurity practices on selected productive performance indicators was estimated.

### Farm selection

The Teagasc ePM is a herd monitoring system available on a voluntary basis to all the farmers in the Republic of Ireland. In 2016, it included 129 pig herds representing over 96,000 sows or 65% of the national commercial sow herd. All the farmers providing data to the ePM were offered the biosecurity assessment of their farrow-to-finish farms using Biocheck.UGent™ and 58 farmers participated voluntarily. Farms were recruited through the Teagasc advisory service and represent approximately 29% of the national commercial sow population.

### Biosecurity assessment

Four researchers visited farms and interviewed farmers to complete the Biocheck.UGent™ questionnaire. All interviewers were trained to conduct the questionnaire. The training provides the criteria to frame the questions and provides examples on how to complete the questionnaire given different scenarios to reduce inter-observer variability. A detailed description of the questionnaire and its scores was explained by Backhans et al. [12] and Laanen et al. [5]. Briefly, the questionnaire has 109 closed questions grouped in 12 categories corresponding to either external (six categories) or internal biosecurity (six categories). Each category assesses several practices and its score was given in a rank from 0 (worst scenario) to 100 (best scenario). Each question had a fixed score. External and internal biosecurity scores were computed as a weighted average of the scores achieved in the corresponding categories. Overall biosecurity was computed as the average of external and internal biosecurity scores. A paper copy of the questionnaire was completed at the farm with both farmer and researcher and the results were transcribed to the Biocheck.UGent™ online database and Microsoft Office Excel format. The final scores for each biosecurity category were obtained for each farm and were used for the analysis.

### Performance data

Performance data for the year 2016 were retrieved from the Teagasc ePM database for all 58 farms included in the study. ePM data was collected on farms every trimester with the support of Teagasc advisors and collated into a single database. This information was used to produce the yearly National Pig Herd Performance Report [13], and for different international comparisons like the InterPIG report [14]. The productive performance indicators selected for analysis in the present study were piglet (pre-weaning) and finisher mortality (%), number of pigs produced per sow per year, average daily gain (ADG, g/day) corresponding to the period from weaning to finish, and feed conversion ratio (FCR), corresponding to the same period.

### Statistical analysis

All statistical procedures were performed in R version 3.4.4 (Vienna, Austria, [15]). Alpha level for significance and tendency were 0.05 and 0.10, respectively. Differences in productive performance between the study sample and the Teagasc ePM population were tested by means of independent samples *t* test (means) and F-tests (variance). The effect of biosecurity on productive performance was estimated through multivariable linear models. Productive performance indicators were used as dependent variables and basic farm characteristics not included in the biosecurity score (number of sows, years of experience of farm managers, number of workers, age of the oldest building in which pigs were kept, and age of the youngest building in which pigs were kept), and biosecurity scores were used as independent variables or predictors. First, Spearman Rank correlations were calculated between all the independent variables to detect collinearity. Then, for each performance indicator, a multivariable linear model was fitted with predictors selected from the farm characteristics, and the biosecurity categories’ scores. When fitting the model for piglet mortality, biosecurity scores related to the nursery and finishing unit management were left out. A forward regression approach was used to improve the models fitted (ols_step_forward function from the olsrr package in R [16]), using a cut-off value of 0.15 for predictor retention in the model. Predictors collinearity was further checked using Variance Inflation Criterion (VIF) from the R package rms [17]. Finally, for each model, residuals’ normality was visually assessed. A simple linear model fitting internal biosecurity scores from external scores was also done.

To identify the most relevant biosecurity aspects in Irish pig farms, a Principal Component Analysis (PCA) was also performed on the biosecurity categories and the 2 main dimensions for the principal components were described. After this, Hierarchical Clustering Analysis was used to group farms according to their similarities regarding their internal and external biosecurity practices. Biosecurity scores and productive performance for each cluster were compared by ANOVA followed by Tukey’s correction.

## Results

### Farm characteristics

Table 1 summarizes the characteristics of the 58 pig farms included in this study. The farms employed farm managers with an average of 26.8 ± 10.14 years of experience. The number of sows (hereinafter, average herd size) was strongly correlated to the number of workers on farm (r_s_ = 0.92, *P* < 0.001) with a ratio of one worker per 154 ± 34.1 sows (range = 55–210). Although the oldest farm buildings in which pigs were kept were on average 35.3 ± 25.46 years old, this figure includes a farm which was 210 years old (range = 5–210). The second oldest farm was 60 years old. The youngest buildings were on average 3.9 ± 5.14 years old with some farms reporting to be building new accommodation at the time (range = 0–25). In this study, 34.5% of the farms reported keeping other animals (cattle) for commercial purposes on the farm grounds. Of those farms, 50% kept beef and 55% kept dairy cattle. One farm kept sheep besides beef. Farm characteristics and herd productive performance (Table 1) did not differ between farms keeping other animals for commercial purposes and farms keeping only pigs, except for ADG (740 ± 57.1 vs. 685 ± 56.3 g/day, respectively; *P* < 0.001).

**Table 1:** Description of the sample of 58 Irish farrow-to-finish pig farms used in the 2016 biosecurity assessment and comparison to the Teagasc database (ePM) population (*n* = 129)

Item	ePM mean ± SD	Study sample^a^	
		mean ± SD	median (Range)
Farm characteristics			
No. of sows	726 ± 610.8	754 ± 554.9	639 (113–2479)
Experience of farm manager, years	–	26.8 ± 10.14	28.0 (5.0–50.0)
Number of workers	–	4.9 ± 3.65	4.0 (1.0–16.0)
Age of the oldest building, years	–	35.3 ± 25.46	32.5 (5.0–210.0)
Age of the youngest building, years	–	3.9 ± 5.14	3.0 (0.0–25.0)
Herd productive performance			
No. of pigs produced per sow per year	25.7 ± 2.30	26.0 ± 2.27	25.8 (18.0–31.2)
Piglet mortality, %	10.5 ± 2.80	10.3 ± 2.70	9.8 (5.1–16.3)
Finisher mortality, %	2.4 ± 1.47	2.2 ± 0.97	2.0 (0.8–5.1)
Average daily gain^b^, g/day	703 ± 79.8	704 ± 62.0	699 (554–856)
Feed conversion ratio^b^	2.41 ± 0.171	2.38 ± 0.144	2.36 (2.01–2.78)

^a^Farm characteristics retrieved from the Biocheck.UGent™ scoring tool which was applied to 58 Irish farrow-to-finish pig farms from February to May 2016. The correspondent herd productive performance was retrieved from the Teagasc ePM for the year 2016

^b^Average daily gain and Feed conversion ratio correspond to the period from weaning to finish

### Farm productive performance

The average herd size of the farms included in the study was 754 sows (range = 113–2479). All the values obtained for productive performance indicators were in the inter-quartile range for the InterPIG report [14], except for piglet mortality which is on the lowest 25%. For ADG, in Ireland it included the period from weaning (29 ± 3.7 d; 7.1 ± 0.46 kg) to finish (107 ± 5.2 kg live-weight). Piglet and finisher mortality showed great variability across farms with a coefficient of variation (CV) of 28.7 and 44.7%, respectively. The number of pigs produced per sow per year, ADG (g/day), and FCR showed less than 10% variability across farms (CV = 8.7, 8.8, and 5.9%, respectively). Between the study sample and the ePM population, differences were found only in the variance of finisher mortality (%) and ADG (g/day) (*P* > 0.001 and *P =* 0.037, respectively), but not in their means. No other differences regarding means or variance were found (*P* > 0.05) across average herd size, piglet mortality (%), number of pigs per sow per year, or FCR.

### Biosecurity scores

The response rate was 100% for all the questions in the questionnaire. The results of the biosecurity assessment are presented in Table 2. The overall biosecurity score was 68.3 ± 9.52. Total external biosecurity scored higher than internal biosecurity (*P* < 0.001) and its practices were applied consistently across farms (CV = 9.8%). The highest score in this category was achieved in the category purchase of animals and semen (98.8 ± 5.05, range = 70–100). The lowest score in this category was in the feed, water, and equipment supply (54.5 ± 14.57). Regarding internal biosecurity, disease management scored the highest with 82.4 ± 21.55, and cleaning and disinfection obtained the lowest score (42.0 ± 27.25) with 12.1% of the farms not applying any of these practices (score 0).

**Table 2:** Biosecurity scores (Biocheck.UGent™) for the different categories of internal and external biosecurity in 58 farrow-to-finish Irish pig farms

	Mean	SD	Median	Min	Max
External biosecurity score^a^	78.7	7.75	79.0	62.0	94.0
Purchase of animals and semen	98.8	5.05	100.0	70.0	100.0
Transport of animals, removal of manure and dead animals	80.1	11.26	83.0	43.0	96.0
Feed, water, and equipment supply	54.5	14.57	53.0	10.0	80.0
Personnel and visitors	73.9	18.61	76.0	24.0	100.0
Vermin and bird control	68.3	19.84	70.0	30.0	100.0
Environment and region	79.5	23.35	80.0	20.0	100.0
Internal biosecurity score^a^	57.4	14.16	60.0	29.0	80.0
Disease management	82.4	21.55	80.0	20.0	100.0
Farrowing and suckling period management	53.6	18.75	57.0	7.0	86.0
Nursery unit management	63.5	16.11	64.0	36.0	100.0
Fattening unit management	72.7	22.12	79.0	21.0	93.0
Measures between compartments and use of equipment	50.0	16.16	50.0	21.0	86.0
Cleaning and disinfection	42.0	27.25	40.5	0	95.0
Overall biosecurity score	68.3	9.52	70.0	47.0	87.0

^a^Biosecurity scores are computed from the practices assessed in each category. Category scores are given in a rank from 0 (worst scenario) to 100 (best scenario). External and internal biosecurity scores correspond to the average of the scores obtained in the corresponding categories. The overall biosecurity corresponds to the average between the external biosecurity score and the internal biosecurity score

### Effect of biosecurity scores and farm characteristics on productive performance

The number of workers was left out of the predictors due to collinearity with average herd size (r_s_ = 0.92, *P* < 0.001). Among the biosecurity categories, the purchase of animals and semen was also left out of the predictors due to its low variability (CV = 5.1%). Table 3 summarizes the models selected.

**Table 3:** Multivariable linear regression modelling of herd productive performance^a^

Outcome	Predictor	Estimate	SE	*P*-value
Piglet mortality, %	Intercept	12.04	1.334	< 0.001
Adjusted *R*^2^ = 0.08	Age of the youngest building, years	0.13	0.066	0.067
*P =* 0.039	Score for feed, water, and equipment supply	−0.04	0.023	0.079
Finisher mortality, %	Intercept	1.50	0.683	0.032
Adjusted *R*^2^ = 0.23	No. of sows [per 100 sows]	0.8	0.21	< 0.001
*P =* 0.002	Score for disease management	−0.01	0.006	0.028
	Score for environment and region	0.01	0.005	0.059
	Score for nursery unit management	0.02	0.008	0.050
	Score for measures between compartments and use of equipment	−0.01	0.008	0.126
ADG, g/day	Intercept	706.27	37.734	< 0.001
Adjusted *R*^2^ = 0.16	No. of sows	−0.03	0.0133	0.043
*P =* 0.006	Experience of farm manager, years	−1.65	0.734	0.029
	Score for disease management	0.73	0.343	0.039

^a^Each productive performance indicator (piglet mortality (%), finisher mortality (%), number of pigs per sow per year, ADG (g/day), and FCR) was modelled from herd characteristics and biosecurity scores (categories), presented in Tables 1 and 2, respectively. The table presents the final models after a forward regression approach with a cut-off value of 0.15 for predictor retention. The models fitting the number of pigs per sow per year and FCR were not significant (overall F-test with *P =* 0.067 and *P =* 0.075, respectively)

The model selected for piglet mortality (%) explained 8% of the variability. There was an increase in mortality with age of the youngest building in which pigs were kept (*P* < 0.001), and a tendency for it to decrease in farms with better scores in the biosecurity category referring to feed, water, and equipment supply (*P =* 0.079).

The model for finisher mortality (%) explained 23% of the variability. Mortality increased with the average herd size (*P* < 0.001) and decreased with good disease management scores (*P =* 0.028). High scores in the categories environment and region and in nursery unit management tended to be related to higher mortalities (*P =* 0.059 and *P =* 0.050, respectively). Good measures between compartments and use of equipment seemed to decrease finisher mortality although this was not statistically significant (*P =* 0.126).

The model for ADG (g/day) explained 16% of the variability. It decreased in large farms (No. of sows, *P =* 0.043) and with the experience of the farm manager (*P =* 0.029). Good practices in disease management improved ADG (*P =* 0.039).

### Relationship between internal biosecurity and external biosecurity

Around 20% of the variability in internal biosecurity (adjusted *R*^2^ = 0.20, *P* < 0.001) could be explained by the scores obtained in external biosecurity:

$$ Internal\ biosecurity=-8.434+0.836\ast External\ biosecurity $$

### Farm clusters based on biosecurity practices

**Fig. 1:**
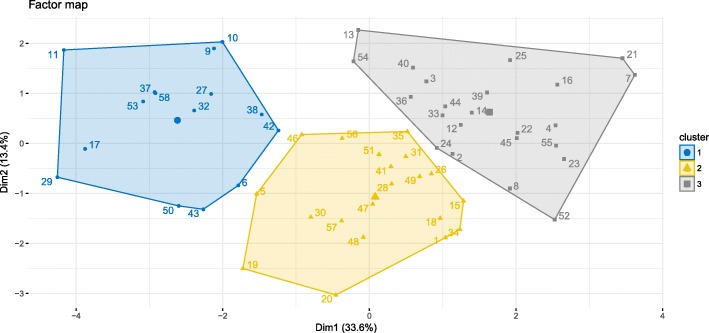
Clusters of farms grouped according to their biosecurity scores in external and internal biosecurity categories. Legend: Dim1 – Dimension 1; Dim2 – Dimension 2. A Principal Components Analysis followed by Hierarchical Clustering Analysis grouped farms according to their scores in external and internal biosecurity practices. Dimension 1 was mainly related with internal biosecurity and dimension 2 was mainly related with external biosecurity. The three clusters identified group farms with low internal biosecurity and high external biosecurity (cluster 1), average internal and external biosecurity (cluster 2), and high internal and external biosecurity (cluster 3)

**Fig. 2:**
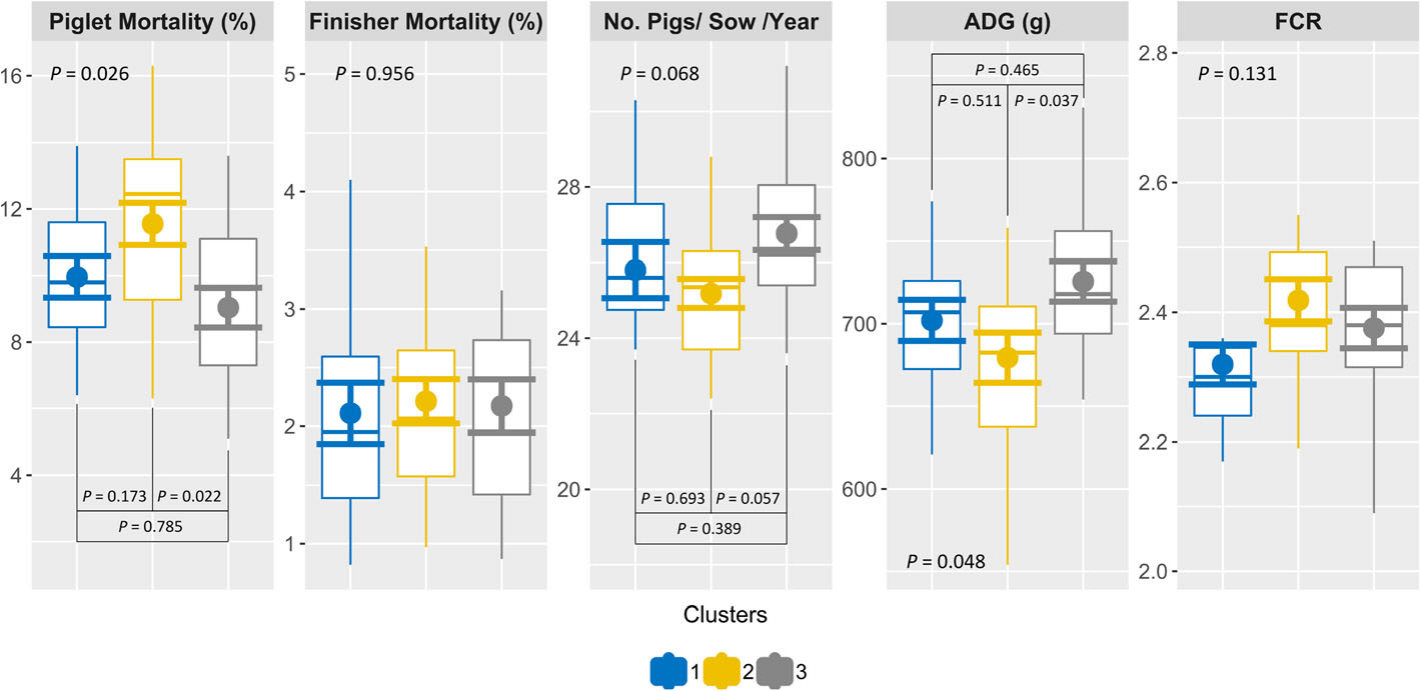
Boxplots of productive performance indicators (with mean ± SD) across farm clusters of farms grouped according to their biosecurity scores in external and internal biosecurity categories. Legend: ADG (g/day) – Average daily gain, FCR – Feed conversion ratio. The clusters represent farms with similar biosecurity scores in external and internal categories. Cluster 1 groups farms with low internal biosecurity and high external biosecurity, cluster 2 groups farms with average external and internal biosecurity, and cluster 3 groups farms with high external and internal biosecurity scores. The productive performance of the farms in each cluster is presented above. ANOVA tests followed by Tukey’s correction were used to test differences in productive performance across those clusters

The first two dimensions of the PCA of the farms depending on their biosecurity practices accounted for 47% of variability. Dimension 1 accounted for 33.6% of variability and was mainly explained (66.1%) by internal biosecurity practices. Dimension 2 accounted for 13.4% of variability and was mainly linked to external biosecurity (68.4%). The main categories contributing to the clustering of the farms were: cleaning and disinfection, compartmentalization, transport of animals and removal of manure and dead animals, and management of the different stages in dimension 1 (mainly internal biosecurity categories); and the environment and region, feed, water, and equipment, management of the different stages, and personnel and visitors in the dimension 2 (mainly external biosecurity categories). Three clusters of farms were identified based on their similarities in biosecurity practices (Fig. 1). The average internal biosecurity score in cluster 1, 2, and 3 was (mean ± SD) 38.4 ± 7.6, 61.4 ± 6.99, and 66.3 ± 9.81, respectively. This score differed statistically between cluster 1 and cluster 2 or 3 (*P* < 0.001). The average external biosecurity score in cluster 1, 2, and 3 was 73.2 ± 5.12, 74.4 ± 5.33, and 86.1 ± 4.47. This score differed statistically between cluster 3 and cluster 1 or 2 (*P* < 0.001). No other differences were found between clusters in regard to internal and external biosecurity scores. The productive performance indicators for each cluster of farms are presented in Fig. 2. Cluster 2 and cluster 3 were different for piglet mortality (11.6 ± 2.84% vs. 9.4 ± 2.39%, *P =* 0.022), and ADG (679 ± 68.2 g/day vs. 726 ± 58.3 g/day, *P =* 0.037). Cluster 2 and 3 tended to be different in the number of pigs per sow per year (25.2 ± 1.71 vs. 26.8 ± 2.08, *P =* 0.057). Finisher mortality and FCR did not differ between clusters (*P =* 0.956 and *P =* 0.131, respectively).

## Discussion

The aim of this study was to describe biosecurity practices among Irish pig farms and their relationship with productive performance. The overall biosecurity scores agreed to what has been described in Belgium, France, Germany, Sweden, and Denmark [3, 11]. External biosecurity was higher in Ireland than in other countries, except for Denmark whose pig sector is focused on high health with strict biosecurity practices [11]. The Irish pig sector includes mostly closed herds, resulting in less animal movements with 94.5% of the farms reported to buy only semen from genetic companies and not purchasing gilts. In other countries, gilts are purchased and quarantined on farm before introducing them to the herd [3, 18, 19]. This is a risk for external biosecurity not existing in Ireland. However, rearing gilts on site may have negative effects on internal biosecurity that are not included in this study. Internal biosecurity scores showed a lack of compliance with cleaning protocols and compartmentalization within the farm. The structure of the farms in Ireland (farrow-to-finish) and the heterogeneity of the facilities (age of the buildings) contribute to the variability seen in internal biosecurity practices. Many Irish farms have grown in size by adding new buildings to older, but still functional, facilities. This heterogeneity makes standardization of protocols to control diseases like PRRS difficult. It is likely that farmers do not valorise the pertinence of internal biosecurity. Casal et al. [18] states that farmers are likely to implement biosecurity measures they perceive as important. However, the awareness towards biosecurity has traditionally been focused on external biosecurity by farmers to avoid those diseases not endemic to their farms. In recent years, the key importance of internal biosecurity practices to reduce disease and improve profitability has resurged and gained new strength. In any case, internal biosecurity was positively correlated to external biosecurity in accordance with other studies highlighting the inter-relationship between both aspects of biosecurity [3, 10–12].

The multivariable model for pig mortality explained only 8% of the variability. The age of the buildings was directly related to mortality. Although 79% of the farms had built new housing for pigs within the previous 5 years, several farms had their latest renovation 10 or 15 years ago. Piglet mortality was also associated to poor biosecurity on feed, water, and equipment supply. As shown in the case of porcine epidemic diarrhoea, these supplies increase the risk of introduction of new diseases, which can be linked to higher mortality. Surprisingly, the farrowing unit management was not retained in the piglet mortality model. This management, as measured by the Biocheck.UGent™, focuses on cross-fostering practices, disinfection of materials between litters, and castration protocols. Other factors such as sow management, farrowing supervision, colostrum intake, split suckling and training of staff [20, 21] may have a greater impact on piglet mortality than the practices captured in the Biocheck.UGent™.

The model for finisher mortality explained 23% of the variability. Bigger farms had higher finisher mortality. Although in our data the bigger the farm, the higher the number of workers, we suspect that bigger farms may have a greater ratio of pigs per worker with less attention paid to individual finisher pigs, as suggested by Agostini et al. [22]. Some of the workers in bigger farms were many times dedicated to jobs that are externalized in smaller farms like general maintenance or feed manufacturing. This area needs further research to specify the types of staff in pig farms and its effects on health and performance. The size of the farm may also have an effect independent of the number of workers. Gardner, Willeberg and Mousing [23] described the duality faced by bigger farms which, facing higher risks of infection due to frequent animal movements and high pressure of infection but having higher biosecurity standards to minimize those risks. Finally, better disease management, including herd health protocols and veterinary expertise, were linked to decreased mortality. The correlations between finisher mortality and areas with lower pig density and management of the nursery unit seem contradictory and cannot be explained although no confounding effects were found.

As for the ADG model, it explained 16% of the variability. Average herd size and experience of farm managers had a negative impact on ADG, and a better disease management was positively correlated to ADG. The negative impact of herd size in growth rate could be related to the association with finisher mortality. In herds with higher disease pressure, growth rates are decreased [24]. Other factors such as herd health or vaccination protocols may have a role in this association. The negative impact of experience could be related to several factors. Laanen et al. [5] found that older farmers were associated with older infrastructures and poor internal biosecurity which could result in a lesser ability to address production challenges.

The associations found between biosecurity categories and productive performance suggests that, in general, farms with good biosecurity had better performance. Laanen et al. [5] identified such associations with ADG and FCR, but not with finisher mortality. Further similarities between that study and ours are the low R^2^, meaning only a small proportion of the variability of the productive performance was explained by biosecurity practices. Indeed, the Biocheck.UGent™ was, as many other biosecurity assessment tools, designed by expert panels using experience and logical reasoning but not scientific validation to support biosecurity practices [7]. Thus it lacks the baseline factors impacting on performance such as herd health status, genetics, use of antimicrobials and vaccinations, and feeding practices. An alternative to this bias would be to model performance using the practices assessed (individual questions) instead of aggregated scores. Finally, some of these tools were designed to address certain pathogens (i.e. PRRSv), not necessarily providing a risk assessment liable to account for other potentially harmful pathogens [25]. Given the limitations stated above, we used a different approach by grouping the farms according to their biosecurity practices and then comparing their productive performance instead of directly modelling performance.

In a multivariate approach to the data, farms were separated in three clear clusters based on their biosecurity practices. The highest production performance was found in farms from clusters with better external biosecurity but not internal biosecurity. External biosecurity practices are easier to implement (fences, barriers, etc) than internal biosecurity practices (i.e. attitudes and behaviours). The former hint investment and could be linked to better management and the maintenance of health status, which translate into better performance. Internal biosecurity practices, as discussed before, are probably under estimated or not well understood by workers who lack a basic understanding of infectious diseases.

### Limitations of the study

Although this study accounts for almost 30% of all the breeding sows in Ireland, these herds were likely to represent a better end of the Irish pig farms, as suggested by Staaveren et al. [26]. Also, the biosecurity data was collected in a cross-sectional study in in-office interviews which may have led to bias towards answers stating measures believed to be applied on farm rather than stating measures applied [18]. Contributing to this bias was also the different interviewees with farm owners being less likely to be aware of the daily management practices and actual cleaning routines in their farms when compared to farm managers and other workers. The use of the Biocheck.UGent™ tool allowed an easy benchmarking between countries; however it also avoided capturing some variation specific to each national context and may be missing details of important internal biosecurity practices like replacement management or piglet management. Finally, as this was an observational study, causal relationships should not be inferred from the results presented.

## Conclusions

This study assessed biosecurity practices in Irish pig farms and its impact on production performance. Irish farms follow European tendencies with internal biosecurity posing as the biggest liability. Our results suggested that practices related to the environment and region, feed, water, and equipment supply and the management of the different stages, need to be particularly addressed in poor performing farms to improve productivity of the Irish pig sector. Indeed such recommendations should be adapted to the health status of each farm. Further studies on the economic impact of these biosecurity practices in connection to data on herd health, vaccination and feeding strategies are the key to motivate farmers to change their practices.

## Abbreviations

ADG: Average Daily Gain (g/day)
d: Day
ePM: Teagasc e-ProfitMonitor
FCR: Feed Conversion Ratio
g: grams
kg: kilograms
No.: Number
PCA: Principal components analysis
